# Neurocognitive profiles of 22q11.2 and 16p11.2 deletions and duplications

**DOI:** 10.1038/s41380-024-02661-y

**Published:** 2024-07-24

**Authors:** Ruben C. Gur, Carrie E. Bearden, Sebastien Jacquemont, Ann Swillen, Therese van Amelsvoort, Marianne van den Bree, Jacob Vorstman, Jonathan Sebat, Kosha Ruparel, Robert Sean Gallagher, Emily McClellan, Lauren White, Terrence Blaine Crowley, Victoria Giunta, Leila Kushan, Kathleen O’Hora, Jente Verbesselt, Ans Vandensande, Claudia Vingerhoets, Mieke van Haelst, Jessica Hall, Janet Harwood, Samuel J.R.A. Chawner, Nishi Patel, Katrina Palad, Oanh Hong, James Guevara, Charles Olivier Martin, Khadije Jizi, Anne-Marie Bélanger, Stephen W. Scherer, Anne S. Bassett, Donna M. McDonald-McGinn, Raquel E. Gur

**Affiliations:** 1 Lifespan Brain Institute of the Children’s Hospital of Philadelphia (CHOP) and Penn Medicine, University of Pennsylvania, Philadelphia, PA USA; 2 Department of Psychiatry and Biobehavioral Sciences, Semel Institute for Neuroscience and Human Behavior, University of California, Los Angeles, CA USA; 3 Department of Psychology, University of California, Los Angeles, CA USA; 4 Neuroscience Interdepartmental Program, University of California, Los Angeles, CA USA; 5 Department of Pediatrics, University of Montreal, Montreal, QC Canada; 6 Sainte Justine Hospital Research Center, Montreal, QC Canada; 7 Centre for Human Genetics, University Hospital Gasthuisberg and Department of Human Genetics, KU Leuven, Leuven Belgium; 8 Department of Psychiatry & Neuropsychology, Maastricht University, Maastricht, The Netherlands; 9 Centre for Neuropsychiatric Genetics and Genomics Division of Psychological Medicine and Clinical Neurosciences Cardiff, Cardiff, UK; 10 Department of Psychiatry, Program in Genetics and Genome Biology, Research Institute, The Hospital for Sick Children, Toronto, ON Canada; 11 Department of Psychiatry, University of California San Diego, La Jolla, CA USA; 12 Department of Pediatrics, Perelman School of Medicine of the University of Pennsylvania; 22q and You Center, Clinical Genetics Center, and Section of Genetic Counseling, CHOP, Philadelphia, PA USA; 13 Dalglish Family 22q Clinic and Toronto General Hospital Research Institute, University Health Network; Clinical Genetics Research Program and Campbell Family Mental Health Research Institute, Centre for Addiction and Mental Health; Department of Psychiatry, University of Toronto, Toronto, ON Canada; 14 Department of Human Biology and Medical Genetics, Sapienza University, Rome, Italy

**Keywords:** Psychology, Diagnostic markers

## Abstract

Rare recurrent copy number variants (CNVs) at chromosomal loci 22q11.2 and 16p11.2 are genetic disorders with lifespan risk for neuropsychiatric disorders. Microdeletions and duplications are associated with neurocognitive deficits, yet few studies compared these groups using the same measures to address confounding measurement differences. We report a prospective international collaboration applying the same computerized neurocognitive assessment, the Penn Computerized Neurocognitive Battery (CNB), administered in a multi-site study on rare genomic disorders: 22q11.2 deletions (*n* = 492); 22q11.2 duplications (*n* = 106); 16p11.2 deletion (*n* = 117); and 16p11.2 duplications (*n* = 46). Domains examined include executive functions, episodic memory, complex cognition, social cognition, and psychomotor speed. Accuracy and speed for each domain were included as dependent measures in a mixed-model repeated measures analysis. Locus (22q11.2, 16p11.2) and Copy number (deletion/duplication) were grouping factors and Measure (accuracy, speed) and neurocognitive domain were repeated measures factors, with Sex and Site as covariates. We also examined correlation with IQ. We found a significant Locus × Copy number × Domain × Measure interaction (*p* = 0.0004). 22q11.2 deletions were associated with greater performance accuracy deficits than 22q11.2 duplications, while 16p11.2 duplications were associated with greater specific deficits than 16p11.2 deletions. Duplications at both loci were associated with reduced speed compared to deletions. Performance profiles differed among the groups with particularly poor memory performance of the 22q11.2 deletion group while the 16p11.2 duplication group had greatest deficits in complex cognition. Average accuracy on the CNB was moderately correlated with Full Scale IQ. Deletions and duplications of 22q11.2 and 16p11.2 have differential effects on accuracy and speed of neurocognition indicating locus specificity of performance profiles. These profile differences can help inform mechanistic substrates to heterogeneity in presentation and outcome, and can only be established in large-scale international consortia using the same neurocognitive assessment. Future studies could aim to link performance profiles to clinical features and brain function.

## Introduction

The “genetics first” approach has investigated recurrent rare copy number variants (CNVs), such as those associated with chromosome 22q11.2 and 16p11.2, providing evidence of increased risk for neurodevelopmental psychiatric disorders across the lifespan. This line of research builds on individuals diagnosed when presenting clinically for evaluation and care at health care facilities and centers that recruit for research on rare genetic disorders. There are common neurobehavioral features associated with these CNVs that manifest transdiagnostically in Attention Deficit Hyperactivity Disorder, Anxiety Disorders, Mood Disorders, Autism Spectrum Disorders, Schizophrenia and Psychosis Spectrum Disorders [1, 2]. Notably, features of neurodevelopmental psychiatric disorders associated with these CNVs are similar to the presentation and course of some idiopathic (behaviorally defined) neurodevelopmental disorders. Among rare CNVs, 22q11.2 deletion and duplication as well as 16p11.2 deletion and duplication have been examined for developmental psychiatric disorders including cognitive functioning. A survey conducted at the Geisinger Health System reported that 22q11.2 duplication (0.119%) and 16p11.2 deletion (0.078%) were the most prevalent CNVs and were associated with lifelong cognitive and psychiatric disabilities documented in electronic health records [2]. The extent and nature of neurocognitive deficits associated with these deletions and duplications varies, and studies to date have usually examined a single CNV or either deletions or duplications. Furthermore, these studies have used varied quantity and quality of neurocognitive assessments, with most focusing on an “intelligence quotient” (IQ) assessed as part of a clinical or research evaluation.

Notably, most studies on cognitive functioning in these CNVs were during childhood, adolescence or young adulthood (6–25 years). Less is known about cognitive functioning in adults. Investigations that examined only 22q11.2 deletion have documented a high prevalence of learning difficulties and intellectual disabilities (mostly mild-moderate) and a range of neurocognitive deficits [e.g., 3–7]. Longitudinal studies have suggested that these deficits are associated with psychiatric symptoms [8–12], and may drive their exacerbation [13]. These impairments comprise several neurobehavioral domains including executive functions and social functioning [3–15]. They are influenced by environmental factors [16] and are associated with abnormalities in brain maturation [17]. Fewer studies have examined 22q11.2 duplication only [18, 19] but these have likewise reported learning problems and cognitive deficits. In a study comparing deficits between 22q11.2 deletion and 22q11.2 duplication (*n* = 19 in each group), and another larger study (106 22q11.2 deletion, 38 22q11.2 duplication), it was concluded that patients with 22q11.2 duplication have a milder cognitive impairment than the 22q11.2 deletion counterparts [20].

Studies examining 16p11.2 deletion have likewise reported reduced intellectual functioning [21] and neurocognitive deficits partly associated with psychopathology [22–24]. There is also evidence for abnormalities in white matter integrity associated with these deficits [25]. Other studies have compared 16p11.2 deletion with 16p11.2 duplication [26–28] finding similar overall functioning across groups, although variance has been reported to be higher in 16p duplication [29]. A 16p11.2 study of 217 deletion carriers, 77 deletion family controls, 114 duplication carriers, and 32 duplication family controls reported higher frequency of psychotic symptoms in duplication compared to deletion carriers [30]. A study comparing individuals with 16p11.2 deletion to 16p11.2 duplication on structural magnetic resonance imaging and neurocognitive performance (*n* = 79 in each group), reported distinct anatomic abnormalities associated with neurocognitive deficits [31]. A study of 82 individuals with 16p11.2 deletion, 50 with 16p11.2 duplication, 370 with 22q11.2 deletion, and 45 with 22q11.2 duplication reported that autism features were largely comparable across the four groups [32]. Significant variability in IQ was noted in CNVs of both loci.

The conclusions that can be drawn from studies to date are limited, as most were based on small sample sizes and because the extent and granularity of the neurobehavioral measures were variable. Because these CNVs are rare, attaining sufficient sample sizes of individuals for drawing firm conclusions requires multi-site collaborations with harmonized measures across sites. The Genes to Mental Health Network (G2MH) [1] was established for this purpose, to accrue a prospective sample with uniform assessment, implementation protocol, and shared data management and quality control. The present study reports the neurocognitive profile of these four groups (22q11.2 deletion/duplication, 16p11.2p deletion/duplication) based on administration of the same neurocognitive battery across sites. The Penn Computerized Neurocognitive Battery (CNB) offers a neuroscience-based assessment of major behavioral domains linked to brain systems based on functional neuroimaging [11, 33–35]. It provides measures of executive functions, episodic memory, complex cognition, social cognition and psychomotor speed. It has been applied to children, adolescents and adults, including individuals with 22q11.2 deletion [33, 34], where it has been associated with IQ with a moderate intraclass correlation coefficient (ICC = 0.57) [36].

The goal of the present project is to examine the pattern of neurocognitive performance on the CNB in a multisite international collaboration and evaluate gene-dosage effects by comparing genomic variants associated with deletion or duplication in 22q11.2 and 16p11.2 loci on multiple domains related to brain function. Our study was aimed to advance the field by directly comparing these reciprocal CNVs, highly penetrant for developmental neuropsychiatric disorders, using the same neurocognitive battery to assess multiple cognitive domains, and in an unprecedently large sample. Findings of distinct neurocognitive profiles across CNVs, despite broad convergence at the symptom level, suggest that neurocognitive markers may provide a window into distinct underlying brain mechanisms. Based on the extant literature, which indicates greater burden of psychotic symptoms associated with 22q11.2 deletions [20] and 16p11.2 duplications [30], we hypothesized that this pattern will be reflected in the neurocognitive deficit profiles by showing greater relative deficits in 22q11.2 deletions and 16p11.2 duplications. The computerized testing allowed us to examine whether the CNVs differentially affect accuracy or speed. We also examined the association between IQ and the CNB-based estimate of overall performance.

## Materials and methods

### Overview

This multisite international collaborative project – “Dissecting the effects of genomic variants on neurobehavioral dimensions in CNVs enriched for neuropsychiatric disorders” – is one of several projects of the G2MH Network. This project includes seven data collection sites, four in North America (Philadelphia, Los Angeles, Montreal, Toronto), three in Europe (Cardiff, Leuven, Maastricht), and two primary genomic analysis sites (Toronto, San Diego). Here we focus on the prospective data collection of the CNB, describing procedures and results from the current sample.

### Study participants

The current sample includes 761 unrelated individuals with IQ ≥ 40, good quality cognitive data, and a CNV at the 22q11.2 or 16p11.2 locus, confirmed by clinical fluorescence in situ hybridization (FISH), comparative genomic hybridization, single nucleotide polymorphism (SNP) microarray, or multiplex ligation‐dependent probe amplification (MLPA) [37]. Participants were recruited from established academic clinical research settings that specialize in the study of rare genetic disorders. IQ estimates were based on chart reviews, and only participants with clinically expected or documented IQ of 40 or higher were invited to participate. IQ test scores were available on 467 of the participants and included WAIS-3 (*n* = 113), WAIS-4 (*n* = 14), WASI-1 (*n* = 86), WASI-2 (*n* = 102), WISC-3 (*n* = 31), WISC-4 (*n* = 40), WISC-5 (*n* = 67), WPPSI-4 (*n* = 14). The average interval between date of participation in the current study and date of IQ testing was 3.12 (SD = 4.92) years (range 0 to 26 years).

#### Inclusion criteria

1. Participants enrolled are aged ≥7 years old. The age range was selected to enable a multifaceted examination of behavioral dimensions and disorders at different settings including home, school, and the community. 2. Able to provide signed informed consent or assent, depending on age and functional ability. 3. Medically stable and able to participate in the evaluation. 4. A sample of blood or saliva is available for deoxyribonucleic acid (DNA) extraction for genomic studies.

#### Exclusion criteria

Potential participants are excluded if they have any of the following conditions that may affect participation and interpretability of data obtained: 1. Medical or neurological disorders that may substantially affect brain function (e.g., untreatable seizures, significant head trauma, central nervous system (CNS) tumor, infection), or visual or auditory limitations (e.g., blindness, deafness). 2. Substance abuse in the past month. 3. Substance dependence not in remission for the past six months. 4. Estimated IQ < 40.

**Fig. 1:**
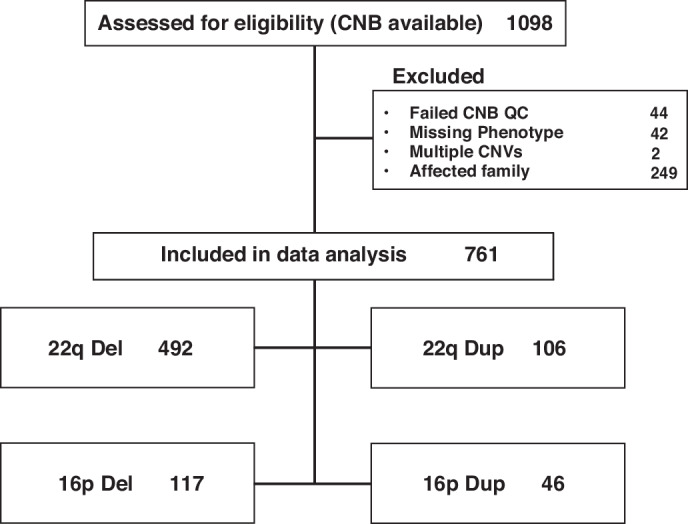
Consort diagram of sample with computerized neurocognitive battery (CNB) data. Top box shows the total sample considered for inclusion in the analysis, the next level shows numbers of participants excluded and reasons for exclusion, and the bottom boxes show sample distribution in the four groups. QC Quality control.

Figure 1 presents a consort diagram of participants with CNVs at the specified loci who were evaluated with the CNB across all recruitment sites and the reasons for exclusion from the current sample. Notably, we included in the current analysis one proband per family when more than one family member had the specified CNV and excluded individuals with CNVs additional to the specified loci.

Table 1 presents sample demographic characteristics by 22q11.2 and 16p11.2 loci. As can be seen, the sample for 22q11.2 deletion is the largest, reflecting ongoing collaborations among established centers conducting research with these patients. Females and males are represented across loci and most participants are of European ancestry. Participants’ characteristics were based on self-reports (and/or collateral report), investigators’ observations, and medical records.

**Table 1:** Demographic characteristics of the sample.

	22qDel (*N* = 492)	22qDup (*N* = 106)	16pDel (*N* = 117)	16pDup (*N* = 46)	Total (*N* = 761)
Sex					
F	267 (54.3%)	52 (49.1%)	59 (50.4%)	25 (54.3%)	403 (53.0%)
M	225 (45.7%)	54 (50.9%)	58 (49.6%)	21 (45.7%)	358 (47.0%)
Age					
Mean (SD)	21.7 (10.4)	21.4 (13.4)	17.7 (9.95)	19.0 (11.4)	20.9 (10.9)
Median [Min, Max]	19.0 [9.00, 59.0]	16.0 [8.00, 59.0]	14.0 [9.00, 49.0]	15.0 [9.00, 55.0]	18.0 [8.00, 59.0]
Race					
Black	16 (3.3%)	6 (5.7%)	0 (0%)	1 (2.2%)	23 (3.0%)
Other	47 (9.6%)	10 (9.4%)	7 (6.0%)	1 (2.2%)	65 (8.5%)
White	429 (87.2%)	90 (84.9%)	110 (94.0%)	44 (95.7%)	673 (88.4%)
Education_years					
Mean (SD)	8.15 (3.61)	7.16 (3.57)	7.22 (3.56)	7.92 (2.90)	7.80 (3.58)
Median [Min, Max]	8.00 [2.00, 19.0]	6.00 [2.00, 18.0]	7.00 [0, 18.0)	8.00 [3.00, 14.0]	8.00 [0, 19.0]
FSIQ					
Mean (SD)	78.0 (13.5)	88.0 (17.0)	80.3 (12.7)	76.9 (13.5)	79.6 (14.3)
Median [Min, Max]	76.0 [43.0, 127]	86.0 [54.0., 134]	79.0 [51.0, 111]	79.0 [54.0, 103]	79.0 [43.0, 134]
CNB_IQ					
Mean (SD)	−0.611 (0.553)	−0.274 (0.591)	−0.283 (0.508)	−0.693 (0.949)	−0.506 (0.591)
Median [Min, Max]	−0.573 [−2.28, 0.746]	−0.208 [−1.66, 0.848]	−0.223 [−1.97, 0.515]	−0.679 [−2.84, 0.549]	−0.464 [−2.84, 0.848]

*F* females, *M* males, *SD* standard deviation, *Min* minimal value, *Max* maximal value, *FSIQ* full scale intelligence quotient, *CNB_IQ* average zscore for accuracy across CNB tests, *Del* deletion, *Dup* duplication

### Procedures

#### Neurocognitive assessment

The Penn CNB [33–35] is a 1-hour computerized battery assessing in the current study five domains across 12 tests: Executive functions (abstraction & mental-flexibility, attention, and working memory); Episodic memory (facial and spatial memory); Complex cognition (nonverbal reasoning and spatial processing); Social cognition (emotion identification, emotion differentiation, and age differentiation); Psychomotor speed (motor speed and sensorimotor speed). Each test provides measures of both accuracy (number of correct responses) and speed (median time for correct responses), except Psychomotor processing tests that provide only speed measures. All responses were made on a keyboard and response time (RT) in milliseconds for each response was recorded. Speed is keyed such that higher values indicate faster performance (RTzscore *-1), and efficiency scores are calculated by averaging the standardized accuracy and speed scores of each test. Notably, we did not include in this study any language tasks (word memory and verbal reasoning) because equating for frequency of words and comparability of linguistic analogies in different languages requires additional steps - such as incorporating input from linguistic analyses using local corpora, adjusting to local dialect and vernacular, and establishing cultural acceptability - to ensure validity of tasks.

#### Implementation

Several steps were taken before starting data collection to ensure that high quality data are obtained in a consistent manner across sites.

#### Translation

The Penn CNB, established in English, has been translated into multiple languages. For the present study, French or Dutch versions were administered by the Montreal, Toronto, Leuven, and Maastricht sites. The validated translation process included professional translation of the initial version followed by back-translation and discussion with the local teams to assure acceptability, following procedures established in other translations of the CNB.

#### Training

All clinical coordinators proctoring the CNB were trained with established procedures. These include a training video providing background on testing and describing each test and the required proctor involvement. This video was followed by a quiz requiring a passing grade of 90%. Next, the trainee administered the CNB to an individual and sent the recorded administration to Penn. Feedback was then provided and additional recordings requested if needed for certification.

#### In-person and remote assessment

At the initiation of the study, all CNBs were administered in-person at the clinical research facilities of each site or at home. With restrictions posed by the COVID-19 pandemic, the Penn CNB team developed and implemented procedures for remote administration. The procedures for remote administration of the CNB followed those of in-person administration [35, 36], with certified test administrators proctoring the tests and ensuring a quiet, private setting at participants’ locations. Proctors underwent training on remote assessments, including familiarity with trouble-shooting the remote platform (i.e., Zoom: https://zoom.us). To complete the virtual CNB, administrators provided participants a unique webpage link and participants were instructed to share their screen with the proctor, so that their performance can be monitored in real time. Through the screen share, the proctor dictated all instructions and observed the participant’s responses for each task. For younger individuals, a parent was present before the assessment started and remained available if needed, but in all cases and for all tasks participants entered the responses themselves. No differences were found between in-person and remote administration modes in studies using the CNB [38].

#### Data quality control (QC)

This step involved a rigorous validation process that used three methods. First, validation codes from the trained test assessors proctoring the CNB indicated when the quality of data was unusable (e.g., participant not engaged or stopped performing task). Second, Penn CNB auto-validation rules were implemented. These are hard-coded, test-specific rules developed to protect against poor data quality that can result from several factors (e.g., unreasonably short response times, unusual repetition of same-key response, unmotivated responding, intentional poor performance, fatigue, etc.). Recently, a third approach that uses data-driven performance validity metrics was also calculated for all tests except for abstraction and mental flexibility and motor speed tests. Data were excluded from analyses if the test was flagged on two or more of the above methods without removing the entire session, such that an individual could have data for some tests but not others. Data was winsorized followed by imputation using the random forest procedure before averaging accuracy or speed [39]. Of the 1098 participants with the identified loci and CNB data, 44 (4%) were excluded from analysis due to failing QC. This proportion is similar to other studies that have used the CNB. Of the 761 participants with valid CNBs, 399 were done in person and 362 remotely; 475 participants (62%) took the battery in English, 210 (28%) in Dutch and 76 (10%) in French. Healthy controls who were administered the CNB at Penn under the same procedures as the CNVs carriers provided normative data across the age range and were balanced for sex. They were medically and psychiatrically assessed and were free of disorders that may impact cognitive performance [33–35].

### Statistical analysis

All analyses were performed using R v4.3.3 (R Core Team, 2024) and SAS software, version 9.4 (SAS Institute, Inc.; Cary, NC). The accuracy and speed scores on the tests were z-transformed using the normative means and standard deviations from the balanced sample of healthy controls. These z-scores, adjusted for Linear and Nonlinear age effects, served as the dependent measures in a Mixed Model Repeated Measures (MMRM) analyses with Locus (22q11.2 vs. 16p11.2) and Deletion vs. Duplication as between-group factors and Test and Measure (accuracy, speed) as the repeated-measures (within group) factors. The Test (Domain) vector included the 10 tests that provided accuracy and speed scores and two tests that provided only speed scores. Sex and Site were entered as covariates. We recognize that sex is an important biological variable, but the sample size is still insufficiently powered across groups to examine five-way interactions with adding Sex as another grouping factor. Significant interactions (two-way, three-way and four-way) were followed up with *post hoc* tests using least square means. We also examined Pearson product moment correlations and ICCs between the CNB performance and IQ measures available in the database. Since these were hypothesis-driven sequential analyses planned for significant interaction effects, no corrections were made for multiple comparisons. The MMRM model was implemented in SAS Mixed procedure, using the Unstructured Covariance Structure option. Type3 tests results were reported. Plots in Figures were produced with R ggplot2. All code is freely and publicly available online (https://github.com/upenn/G2MH/).

## Results

### Locus and deletion vs. duplication effects

Results of the Locus (22q11.2, 16p11.2) × Deletion-Duplication × Measure (accuracy vs. speed) × Domain (neurocognitive test) MMRM are presented in Table 2. As can be seen, several two-way and three-way interactions were significant and, most importantly, there was a significant four-way interaction (*p* = 0.0004), indicating that deletions and duplications in the two loci differentially affect accuracy and speed of neurocognitive performance profiles associated with CNVs.

**Table 2:** Results of the four-way Mixed Model analysis on accuracy and speed scores^a^.

Effect	Num	Den	*F*	*P*
	DF	DF		
Locus	1	1485	1.63	0.2016
Del_Dup	1	1445	0.81	0.3669
Locus*Del_Dup	**1**	**1432**	**12.77**	**0.0004**
Domain	11	1392	0.74	0.7033
Locus*Domain	11	1392	0.55	0.8702
Del_Dup*Domain	**11**	**1392**	**3.08**	**0.0004**
Locus*Del_Dup*Domain	**11**	**1392**	**2.17**	**0.0137**
Acc_Spd	**1**	**1323**	**10.52**	**0.0012**
Locus*Acc_Spd	1	1323	0.01	0.9172
Del_Dup*Acc_Spd	**1**	**1323**	**12.67**	**0.0004**
Del_Dup*Locus*Acc_Spd	**1**	**1323**	**6.39**	**0.0116**
Domain*Acc_Spd	9	1359	1.3	0.2325
Locus*Domain*Acc_Spd	**9**	**1359**	**2.13**	**0.0245**
Del_Dup*Domain*Acc_Spd	9	1359	1.83	0.0587
Del_Dup*Locus*Domain*Acc_Spd	**9**	**1359**	**3.42**	**0.0004**
Sex	1	1338	7.58	0.006
Site	6	1338	0.90	0.4936

Significant effects are in bold.

*Del* deletion, *Dup* duplication, *Num* numerator, *Den* denominator, *DF* degrees of freedom, *P* probability.

^a^Locus = 22q11.2, 16p11.2.

**Fig. 2:**
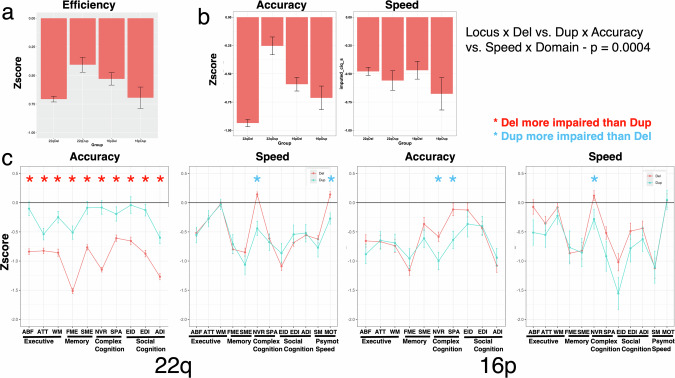
Neurocognitive performance of the groups by Locus and deletion vs. duplication status. **a** Means (+/−SEM) of performance of the four groups in Efficiency (average of accuracy and speed) averaged across tests. **b** Accuracy (left panel) and Speed (right panel) averaged across tests. **c** Neurocognitive profiles of the four groups, 22q11.2 on the left panels and 16p11.2 on the right panels, showing means (+/−SEM) of performance for accuracy on the 10 tests and speed on 12 tests. ^a^Del deletion, Dup duplication, ABF abstraction and mental flexibility, ATT attention, WM working memory, FME face memory, SME spatial (shape) memory, NVR nonverbal (matrix) reasoning, SPA spatial processing, EID emotion identification, EDI emotion intensity differentiation, ADI age differentiation, SM sensorimotor speed, MOT motor speed, Psymot Psychomotor.

The two-way (Locus × Deletion-Duplication), three-way (Locus × Deletion-Duplication × Accuracy vs. Speed) and four-way interactions (the Locus × Deletion-Duplication × Accuracy vs. Speed × Test domain) are shown in Fig. 2 (a, b, and c, respectively). Means of the four Locus × Deletion-Duplication groups on all neurocognitive domains are shown in Supplementary Table S1 along with the statistical comparisons by test.

As can be seen in Fig. 2a, the 22q11.2 deletion group was more impaired in average performance efficiency (average of accuracy and speed) than the 22q11.2 duplication group while the reverse was the case for the 16p11.2 locus, where performance of the duplication group was lower than that of the deletion group. This pattern was seen for accuracy, with the deletion group more impaired than the duplication group for the 22q11.2 locus, while the duplication group showing lower scores than the deletion group for the 16p11.2 locus. For speed, both duplication groups showed lower performance than their deletion counterparts (Fig. 2b). The four-way interaction, displaying group effects for accuracy and speed by test Domain, is illustrated in Fig. 2c (see also box-and-whisker plots in Supplementary Figs. S1 and S2, and means and statistical comparisons in Supplementary Table S1). For the 22q11.2 locus, the deletion group was more impaired than the duplication group on accuracy across tests, but especially in memory and non-verbal reasoning. Speed was comparable for the two groups across tests, except for slower speed in the context of better accuracy for the 22q11.2 duplication group in non-verbal reasoning, perhaps indicating a speed-accuracy tradeoff. Motor speed was also slower in the 22q11.2 duplication compared to the 22q11.2 deletion group. In contrast to the 22q11.2 locus groups, the 16p11.2 duplication group showed greater impairment than the 16p11.2 deletion group on specific neurocognitive domains. This effect was evident for accuracy in complex cognition, both non-verbal (matrix) reasoning and spatial processing domains. For speed, 16p11.2 duplication group showed reduced performance on the complex cognition test of nonverbal reasoning (marginally lower for spatial processing), and they were marginally slower on the social cognition test of emotion identification. Across accuracy and speed, the 16p11.2 duplication group was specifically impaired relative to the 16p11.2 deletion group on complex cognition.

### Association with IQ

**Fig. 3:**
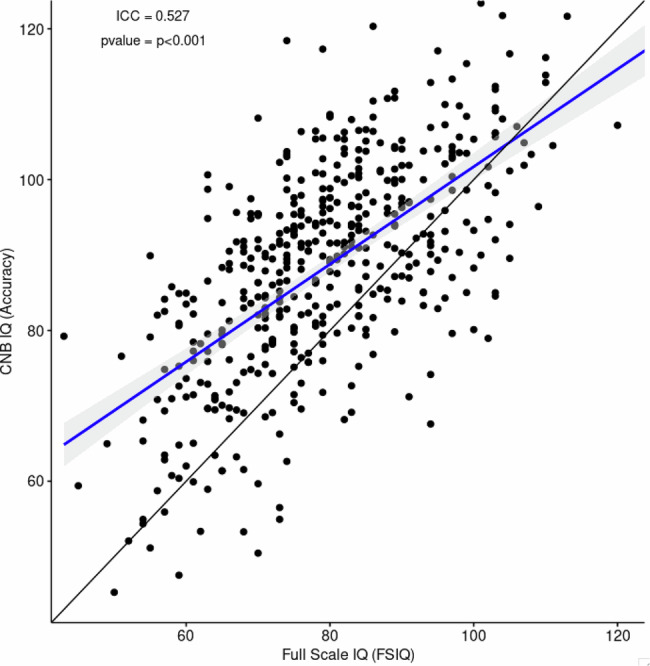
Scatterplot showing the association between full-scale IQ in records and IQ scaled scores based on the average performance accuracy on the CNB.

To allow better bridging of our CNB findings with available literature where an IQ measure has most frequently been used to assess cognitive capacity, we evaluated the association between the CNB estimate of overall accuracy, defined as the average accuracy z-scores across tests, and IQ data available from the participants’ health or research records indicating Wechsler scales scores. The association between the two measures (Fig. 3) is moderate, r(518) = 0.643, ICC = 0.527, *p* < 0.001. The computerized estimates consistently higher than the paper-and-pencil based measures at the lower ranges of performance, as indicated by the majority of observations above the identity line. The partial correlation between the measures was 0.629 after partialling out the interval between the measures, a marginal reduction. This association was consistent across our groups (Supplementary Fig. S3).

## Discussion

The adverse effects of CNVs on cognitive performance have been documented in the literature in studies that used a range of different measures. Most commonly an “intelligent quotient” (IQ) has been used based on standardized clinical tests (e.g., Wechsler scales). More extensive neurocognitive assessments have been reported in clinical and research samples that included examination of both deletions and duplications of CNVs such as 22q11.2 and 16p11.2 [31, 32]. However, currently published reports do not permit comparing locus effects on a range of neurocognitive domains with the same detailed measurements in both deletion and duplication. The present study addresses this gap by reporting results of a comprehensive neurocognitive evaluation of a large multi-site sample of individuals with deletion or duplications at the 22q11.2 or 16p11.2 loci. The computerized neurocognitive battery (CNB) is an extensively validated and efficient instrument that is based on cognitive neuroscience and provides information on accuracy and speed of performance in neurocognitive domains related to brain systems [40]. The data in the current study were collected prospectively following rigorous training and quality assurance procedures. The administration was well tolerated by participants across sites, as evident in the low percentage of quality control rejections (4%).

The overall results support our hypothesis by indicating that whereas deletions are more deleterious than duplications at the 22q11.2 locus, opposite effects were seen at the 16p11.2 locus, where duplications were more deleterious than deletions. Our findings for the 22q11.2 locus are consistent with earlier studies showing that the cognition effects of the deletion are more deleterious than those of the duplication [20]. The literature on 16p11.2 is less consistent [30–32] and our study clarifies that overall, for this locus, the deletion is less deleterious than the duplication with respect to cognitive performance. Notably, the CNVs associated with schizophrenia risk, 22q11.2 deletion and 16p11.2 duplication [20, 30, 41], show greater deficits than the other groups on complex cognition and social cognition.

The computerized format allows separate evaluations of accuracy and speed of performance, and our analysis revealed further specific differences among the groups. At both 22q11.2 and 16p11.2 loci, deletions and duplications were associated with reduced accuracy and speed. The pattern of performance across domains also differed among the four groups. For the 22q11.2 loci, deletion affected accuracy across nearly all domains, while duplication was associated with milder impairment in accuracy with a similar profile. The 22q11.2 duplication group had slower speed with a similar profile, except for slower performance than the deletion group on non-verbal reasoning and motor speed. For the 16p11.2 loci, deletion and duplication had the same effect on accuracy of performance on most domains, but those with a duplication performed more poorly on the complex cognition domain (nonverbal reasoning and spatial processing). The effects of 16p11.2 loci were more pronounced for speed, where the duplication group performed more slowly than the deletion group on complex cognition tests.

The findings complement Chawner et al.’s study [32] that focused on autism profiles but also examined IQ profiles (Full Scale IQ, Verbal IQ & Performance IQ) in 16p11.2 and 22q11.2 CNV carriers. Chawner et al. did not examine specific neurocognitive domains beyond IQ. Although the work combined data from multiple international sites, it was not a pre-planned international effort, and as a result there was no opportunity for cross-site reliability and administration training. The work presented here advances the field because the collection of data across different international sites was prospectively planned and every site used the CNB for cognitive assessment. This meant that a) all sites were trained to administer the CNB using the same protocol, b) regular meetings took place to ensure harmonized data collection across sites c) the same data QC procedures were applied across sites. Furthermore, the CNB provides detailed assessment across multiple specific neurocognitive domains, advancing knowledge on the wide-ranging cognitive impacts of 22q11.2 and 16p11.2 CNVs. Assessment of such neurocognitive domains provides insight into which brain regions and pathways may be disrupted by these CNVs, in comparison to IQ which is a global cognitive measure. Importantly, specific neurocognitive domains are likely to represent focused targets for intervention through cognitive training, in contrast to IQ which is less ameliorable through intervention. Thus, deficits in domains such as attention, working memory and episodic memory offer very specific targets for interventions with quantifiable milestones for effectiveness. Improvement in such domains can positively affect functioning. For example, improved working memory significantly mediated improved adaptive functioning in a longitudinal study of 22q11.2 deletions [13].

Our results can inform common and differing mechanisms through which CNVs may impact cognition and brain function. Such investigations could link individual differences in performance with brain structural and functional parameters to allow individual characterization. For example, a multimodal MRI study of 22q11.2 deletion syndrome showed that brain parameters related to primary visual processing and insular function were relatively intact in individuals with the deletion, while those related to motor feedback, face processing, and emotional memory processes were more impaired compared to controls. Such approaches may help inform potential intervention targets and enhance the specificity of neuroimaging and electrophysiological indices related to cognitive dysfunction [42].

The molecular mechanism of cognitive performance deficit is probably different between 22q11.2 deletions and 16p11.2 duplications, as deletion entails loss of function while duplication suggests gain of function and there could be different pathways. Notably, 16p11.2 genes are enriched in pathways of mitogen-activated protein kinases (MAPK) signaling, growth factor signaling and DNA replication [e.g., ref. 43] while 22q11.2 seems to be enriched for synaptic function and neurotransmission among many other factors [e.g., ref. 44]. Mechanistic insights on the neurobehavioral deficits can benefit from preclinical work, which has identified 22q11.2 and 16p11.2 genes that contribute to the accuracy and speed of social and cognitive functions in mice (e.g., refs. 45, 46). Notably, animal models and human studies examining human induced pluripotent stem cells (iPSCs) have focused on deletions [45–48]. Further mechanistic insights on how CNVs affect behavior could be gleaned by developing and evaluating effects of duplications in animal models and human iPSC studies.

As most previous studies have examined IQ as a measure of cognition, we related global performance on the computerized neurocognitive battery to IQ measures available in clinical and research records or assessed across the sites. The correlation between these measures was moderate (r = 0.643) in the present study, and the ICC (0.527) slightly lower than an earlier study reporting an ICC of 0.57 between IQ and CNB average accuracy for a sample with 22q11.2 deletions [36]. The association between IQ and average CNB performance is unlikely attenuated by the time difference between the assessment, as suggested by the negligible change in association to 0.629 after partialling out this interval. It is likely further attenuated by the various instruments used for measuring IQ. Notably, the CNB is based on functional neuroimaging and includes domains that are not assessed in IQ tests. As seen in Fig. 3, estimates based on CNB performance are consistently higher than IQ at the lower ability ranges. This pattern is similar to that reported in our earlier study of 22q11.2 Deletion participants [36]. A possible reason for this finding is that a low IQ score could mask relative strengths in specific neurocognitive domains that are not measured by IQ tests. Alternatively, the computerized testing format is more game-like, thereby boosting motivation, and gives equal weight to accuracy and speed, allowing individual trade-offs.

### Limitations

While we report the results of a large collaborative study for rare CNVs, the sample size across the loci varies. Most participants are of European ancestry, reflecting location of sites and perhaps these CNVs being underdiagnosed in other ancestries. This issue needs further investigation. Differential ascertainment for variants is another potential limitation. For example, individuals with 22q11.2 deletions are more likely to be referred for testing for physical issues such as congenital heart defects, whereas those with 22q11.2 duplications are more likely to be referred for developmental reasons. The healthy controls for standardizing performance were from the University of Pennsylvania normative database, as collection of normative controls was not part of the funded study. A more rigorous approach would have entailed collection of normative samples at each site. However, there is evidence that normative data from the University of Pennsylvania are comparable to other settings [6, 36]. Furthermore, the age range across the loci was broad and sample sizes limited systematic examination of age bins, which can be performed in the future with larger samples. A more comprehensive analysis of IQ data is beyond the scope of this report. We are not reporting here on breakpoints in the CNV loci, as this information will be part of the whole-genome sequencing that will become available at the conclusion of the study. Similarly, the association of neurocognitive performance with neuropsychiatric disorders, which are common in these CNVs, medications, and other medical conditions will be examined as part of the next phase of the study.

Notwithstanding these limitations, our study demonstrates that efficient prospective measures can help identify differential CNV effects on neurocognition. Our approach offers a unique opportunity to characterize the functional consequences of genetic mutations highly penetrant for multiple neuropsychiatric disorders. They have implications for genotype-phenotype relationships in psychiatry. Different psychiatric risk variants result in different cognitive profiles and perhaps trajectories and may represent different pathways to psychiatric outcomes. Examining cognitive endophenotypes provides a step further in understanding the route from genomic risk to psychiatric outcomes. Future studies could further elucidate unique and common features associated with these and other CNVs, pointing to mechanistic links between genomic variations and their phenomic manifestations.

## Supplementary information

Supplementary Table S1

Supplementary Figures

## Data Availability

This dataset will be available through the National Data Archive (https://nda.nih.gov), including a data dictionary post data completion and data release.
