# Supplementary material for: Neurocognitive profiles of 22q11.2 and 16p11.2 deletions and duplications
Source: Mol Psychiatry. 2024 Jul 24;30(2):379–87. doi: 10.1038/s41380-024-02661-y (PMC11746132; doi:10.1038/s41380-024-02661-y)
Supplement: Supplementary file 1 — Supplementary Table S1 [file 41380_2024_2661_MOESM1_ESM.pdf]

	ACCURACY												SPEED											
Group	22qDel		22qDup				16pDel		16pDup				22qDel		22qDup				16pDel		16pDup			
	Mean	SD	Mean	SD	t	P	Mean	SD	Mean	SD	t	P	Mean	SD	Mean	SD	t	P	Mean	SD	Mean	SD	t	P
DOMAIN	N=492		N=106		DF=596		N=117		N=46		DF=161		N=492		N=106		DF=596		N=117		N=46		DF=161	
ABF	-0.84	1.09	-0.10	1.20	**-5.99**	**<.0001**	-0.65	1.14	-0.88	1.10	<1.5	NS	-0.52	1.49	-0.55	1.38	<1.5	NS	-0.07	1.36	-0.51	1.47	1.80	0.0737
ATT	-0.82	1.04	-0.54	1.01	**-2.08**	**0.038**	-0.67	1.21	-0.65	1.02	<1.5	NS	-0.28	1.26	-0.27	1.40	<1.5	NS	-0.36	1.36	-0.55	1.45	<1.5	NS
WM	-0.86	1.35	-0.25	1.02	**-4.15**	**<.0001**	-0.74	1.23	-0.69	1.02	<1.5	NS	-0.02	1.02	-0.03	0.90	<1.5	NS	-0.08	0.82	-0.22	0.98	<1.5	NS
FME	-1.51	0.97	-0.51	1.17	**-8.90**	**<.0001**	-1.16	0.91	-0.96	1.15	<1.5	NS	-0.80	1.69	-0.71	1.68	<1.5	NS	-0.87	1.65	-0.77	1.91	<1.5	NS
SME	-0.77	1.00	-0.09	1.13	**-6.35**	**<.0001**	-0.37	1.25	-0.61	1.07	<1.5	NS	-0.85	1.62	-1.06	1.68	1.68	0.0936	-0.82	1.53	-0.85	1.64	<1.5	NS
NVR	-1.15	0.89	-0.08	1.18	**-10.35**	**<.0001**	-0.58	0.87	-1.00	1.02	**2.71**	**0.0075**	0.14	0.95	-0.44	1.26	**5.24**	**<.0001**	0.12	0.93	-0.28	1.15	**2.30**	**0.0226**
SPA	-0.61	1.20	-0.19	1.25	**-3.13**	**0.0018**	-0.12	1.16	-0.64	1.00	**2.64**	**0.0090**	-0.61	1.36	-0.68	1.47	<1.5	NS	-0.52	1.17	-0.92	1.75	1.69	0.0929
EID	-0.66	1.45	-0.04	1.47	**-4.06**	**<.0001**	-0.13	1.30	-0.37	1.51	<1.5	NS	-1.09	1.60	-0.87	1.72	<1.5	NS	-1.02	1.60	-1.56	1.86	1.83	0.0693
EDI	-0.87	1.23	-0.13	1.04	**-5.09**	**<.0001**	-0.43	1.16	-0.40	1.11	<1.5	NS	-0.68	1.36	-0.54	1.64	<1.5	NS	-0.49	1.49	-0.78	1.59	<1.5	NS
ADI	-1.27	1.15	-0.60	1.04	**-5.02**	**<.0001**	-1.08	1.24	-0.94	1.04	<1.5	NS	-0.56	1.18	-0.52	1.56	<1.5	NS	-0.44	1.35	-0.63	1.42	<1.5	NS
SM													-0.62	1.49	-0.77	1.63	<1.5	NS	-1.12	1.85	-1.11	1.86	<1.5	NS
MOT													0.14	1.15	-0.27	0.98	**4.38**	**<.0001**	0.04	1.22	0.05	1.14	<1.5	NS
