# Supplementary material for: Neurocognitive profiles of 22q11.2 and 16p11.2 deletions and duplications
Source: Mol Psychiatry. 2024 Jul 24;30(2):379–87. doi: 10.1038/s41380-024-02661-y (PMC11746132; doi:10.1038/s41380-024-02661-y)
Supplement: Supplementary file 2 — Supplementary Figures [file 41380_2024_2661_MOESM2_ESM.docx]

**Supplementary Figures**

Figure S1. Box-and-whisker plots for the four groups showing accuracy of performance on the individual CNB tests.

**

Figure S2. Box-and-whisker plots for the four groups showing speed of performance on the individual CNB tests.

**

Figure S3. Scatterplot showing the association between full-scale IQ in records and IQ scaled scores based on the average performance accuracy on the CNB in the four groups.
